# The role of family factors in antiretroviral therapy (ART) adherence self-efficacy among HIV-infected adolescents in southern Uganda

**DOI:** 10.1186/s12889-020-8361-1

**Published:** 2020-03-17

**Authors:** Proscovia Nabunya, Ozge Sensoy Bahar, Bin Chen, Daji Dvalishvili, Christopher Damulira, Fred M. Ssewamala

**Affiliations:** 1 Washington University in St. Louis, Brown School of Social Work, One Brookings Drive, St. Louis, MO 63130 USA; 2 Washington University in St. Louis, St. Louis, MO USA; 3 International Center for Child Health and Development, Masaka, Uganda

**Keywords:** Adherence self-efficacy, HIV-infected adolescents, Family cohesion, Antiretroviral therapy adherence, Sub-Saharan Africa

## Abstract

**Background:**

Adolescents living with HIV in sub-Saharan Africa are a vulnerable group at the intersection of poverty and health disparities. The family is a vital microsystem that provides financial and emotional support to achieve optimal antiretroviral therapy (ART) adherence. In this study, we explore the association between family factors and ART adherence self-efficacy, a significant psychological concept playing a critical role in ART adherence.

**Methods:**

Data from an NIH-funded study called *Suubi + Adherence,* an economic empowerment intervention for HIV positive adolescents (average age = 12.4 years) in southern Uganda was analyzed. We conducted multilevel regression analyses to explore the protective family factors, measured by family cohesion, child-caregiver communication and perceived child-caregiver support, associated with ART adherence self-efficacy.

**Results:**

The average age was 12.4 years and 56.4% of participants were female. The average household size was 5.7 people, with 2.3 children> 18 years. Controlling for sociodemographic and household characteristics, family cohesion (*β* = 0.397*, p* = 0.000) and child-caregiver communication (*β* = 0.118, *p* = 0.026) were significantly associated with adherence self-efficacy to ART.

**Conclusion:**

Findings point to the need to strengthen family cohesion and communication within families if we are to enhance adherence self-efficacy among adolescents living with HIV.

**Trial registration:**

This trial was registered with ClinicalTrials.gov (registration number: NCT01790373) on 13 February 2013.

## Background

Uganda is a low-income country with one of the highest prevalence rates of HIV (7.3% among 15–49-year old) worldwide [1]. It is estimated that 130,000 children under the age of 14 in Uganda were living with HIV in 2016 [2]. Although the development of antiretroviral therapy (ART) has made HIV a manageable chronic illness, [3] adherence to ART needs to reach 95% in order to reach the desired treatment outcomes [4, 5]. However, research shows that ART adherence level in Uganda is still low among people living with HIV (PLWH), with only 66% reporting the desired adherence outcomes [6]. Moreover, in rural areas, adherence rates are much lower, with only 55% adhering to their medication [7]. Furthermore, the ART coverage for children in Uganda is estimated to be only 47% of the target population [8]. Yet, low ART adherence can result in increased viral duplication, rapid disease progression, reduced life quality, and even premature mortality [9]. Therefore, suboptimal ART adherence among children in Uganda is an urgent health issue that needs to be addressed. Against this backdrop, this study examines family factors that impact ART adherence self-efficacy among perinatally HIV-infected adolescents (aged 10 to 16 years) in southern Uganda so that more targeted interventions can be put in place to improve ART adherence.

### Medication adherence and self-efficacy

In the context of HIV, ART adherence is defined as the degree to which an individual adheres to taking the prescribed antiretroviral drugs [4]. Observable ART adherence levels depend on a range of factors, [10] including self-efficacy i.e. the person’s perception of their own ability to accomplish a behavioral task, [11] which influences a person’s development or maintenance of a health behavior at the affective, cognitive and motivational levels [12]. More specifically, adherence self-efficacy –defined as the confidence in one’s ability to adhere to treatment plans, has been documented as an important predictor of medication adherence in the treatment of HIV and other medical conditions [13]. For example, an individual who feels able to successfully fulfill medication regimes as prescribed, in addition to following a specified diet, as well as executing recommended lifestyle changes, [14] will be more likely to achieve positive health outcomes. Moreover, individuals tend to be more motivated if they perceive that their actions can be completed [15]. Indeed, adherence self-efficacy has been linked to positive outcomes among patients with hypertension, [16] asthma, [17, 18] diabetes [19, 20] pain management [21] and depression [22].

Among PLWH, self-efficacy has been corelated with adherence outcomes [23–29]. For example, a meta-analysis examining the predictors and correlates of ART adherence found that adherence self-efficacy was positively associated with the initiation and maintenance of ART adherence [23]. In another study in South Africa, a strong association between self-efficacy and ART adherence was found in the nonadherent participants, explaining 9.8% of the variance [24]. In the United States and Puerto Rico, Nokes and colleagues [25] found adherence self-efficacy to be a robust predictor of ART adherence and a mediator between environmental influences and cognitive or personal factors. However, few of these studies have been conducted in SSA, and none have focused on children and adolescents living with HIV [24]. Yet, for children who depend on their caregivers to meet their adherence expectations, it is important to examine and understand their cognitive and motivational influences affecting medication adherence, including self-efficacy.

### Social support and adherence

Several studies have documented the role of social support, especially from family members in influencing adherence outcomes [30–38]. For example, a meta-analysis of studies examining social support and patient adherence to medication showed that ART adherence was 1.74 times higher in patients from cohesive families and 1.53 times lower in patients from families experiencing conflict [30]. In Uganda, family cohesion and social support from caregivers/family were associated with self-reported adherence to ART among HIV-infected adolescents [31]. In another study examining the benefits of family and social relationships for health and mental health of PLWH, family functioning significantly contributed to ART adherence and quality of life [32]. Thus, strengthening positive family support and minimizing negative family interactions are crucial for increasing adherence rates [33].

Despite the unique developmental needs of children and adolescents, few studies have specifically focused on family systems among HIV-infected children and adolescents in SSA [31, 34, 35]. Moreover, these studies do not explicitly explore the relationship between family support and adherence self-efficacy –two factors that are critical to HIV management and adherence to treatment protocols among PLWH, including children and adolescents [36]. Thus, to bridge this gap, this study examines whether family factors, such as family cohesion, child–caregiver communication, and perceived child-caregiver support, are associated with ART adherence self-efficacy among HIV-infected adolescents in southern Uganda. We hypothesize that these family factors will be positively associated with positive ART adherence self-efficacy levels over time.

## Methods

### Study sample and context

This study utilized data from the Suubi+Adherence study, a longitudinal randomized clinical trial funded by the National Institute for Child Health and Human Development (Grant # R01HD074949). The Suubi+Adherence study examined an innovative family-based economic empowerment intervention on ART adherence among perinatally HIV-infected adolescents in southern Uganda, a region heavily affected by HIV/AIDS. Uganda has a national HIV prevalence rate of 7.3% among adults aged 15–49, with higher prevalence rates of 12% in the southern region where the study was implemented [1]. The Suubi+Adherence study followed 702 HIV-positive adolescents (306 boys and 396 girls) between 2012 and 2017. Participants were identified and recruited from health clinics in the greater Masaka region associated with Reach the Youth (RTY) Uganda and the Masaka Diocese (our collaborating partners). All health clinics were accredited by the Uganda Ministry of Health to provide ART to all adolescents living with HIV in the region. The inclusion criteria for these adolescents were: 1) 10–16 years old, 2) HIV-positive and know their HIV status, 3) prescribed antiretroviral therapy, 4) living within a family, not an institution, and 5) enrolled in one of the 39 health centers or clinics in Rakai, Masaka, Lwengo, Lyantonde, Bukomasimbi, and Kalungu Districts in the study area. Detailed information on participants recruitment and selection process, as well as the study intervention is described in the study protocol and in our other publications [37, 38].

### Data collection

Data were collected using a 90-min interviewer administered survey at baseline, 12, 24, 36 and 48-months post baseline. Survey instruments and all research related documents (including consent/assent forms) were translated into Luganda – the most widely spoken language in the study region – and back translated into English to ensure accuracy. This process was overseen by certified language experts at the Makerere University in Uganda. All interviewers completed the Collaborative Institutional Training Initiative (CITI) certificate and NIH certificate for protection of research participants, prior to participant contact. In addition, the study team received training on Good Clinical Practices (GCP) so that sensitive research activities were handled appropriately. Details on data protection are provided in the study protocol [37].

### Measures

The dependent variable in this study is adherence self-efficacy, assessed by the HIV Treatment Adherence Self-Efficacy Scale [13]. The scale measures adolescents’ confidence in integrating prescribed ART plan into their routine life and maintaining optimal adherence levels. The scale was previously tested among people living with HIV in the southern region of Uganda, with a high reliability score (Cronbach’s alpha = .92) [39]. There are nine integration questions, such as *“In the past month, how confident have you been that you can stick to your treatment plan even when side effects begin to interference with daily activities?”* and three perseverance questions, such as *“In the past month, how confident have you been that you can continue with the treatment plan your physician prescribed even if your T-cells drop significantly in the next three months?”* The rating range is between 1 and 10, with a higher rating representing higher levels of adherence self-efficacy. The scale has a high reliability (alpha = 0.91).

Family support factors were measured by three indicators: 1) family cohesion, 2) perceived child–caregiver support, and 3) child-caregiver communication, all adapted from the Family Environment Scale, [40] and the Family Assessment Measure [41]. All these measures were tested in our previous studies in Uganda among children affected by HIV/AIDS [31, 42–44].

Family cohesion was assessed by an 8-item scale that measures the degree of commitment, help and support family members provide for one another. Participants were asked to rate how often each item occur in their family, on a 5-point scale (1 = ‘never’ and 5 = ‘always’). Sample items include: “*Family members ask each other for help before asking non-family members.”* and *“Family members like to spend free time with each other.”* The scale had a strong internal consistency (alpha = 0.79). Summary mean scores were created with higher scores indicating high levels of family cohesion.

The perceived child-caregiver support scale assesses social support on two dimensions: (1) acceptance and warmth – the extent to which the child perceives the caregiver as involved in their life; and (2) psychological autonomy – the extent to which the caregiver employs a non-coercive, democratic discipline and encourages the child to express individuality within the family. Participants were asked to rate the adults they live with, on each of the 18 items (range: 18–70, alpha = 0.76), on a 5-point scale (1 = ‘never’ and 5 = ‘always’). Sample items include: *“Child can count on parent/guardian to help in case of a problem,”* and *“Parent/guardian explains why they want the child to do something.”* Summary mean scores were created, with higher scores indicating high levels of perceived child-caregiver support.

The child-caregiver communication scale assesses discussions between the child and the caregiver on 12 items related to issues such as puberty, cigarette smoking, sexual risk taking, puberty, HIV/AIDS, educational plans, etc. Participants were asked to indicate how often they discussed the specific topics with their caregivers, on a 5-point Likert scale (1 = ‘never’ and 5 = ‘always’). Summary mean scores were created with higher scores indicating high levels of child-caregiver communication (range: 10–50, alpha = 0.79).

Control variables included in the analysis were: participants’ gender, age, total number of people in the household, total number of children in the household, primary caregiver (i.e. biological parent, grandparents, and others such as uncle, aunt, brother sister, etc.), school enrollment (whether enrolled in school or not), HIV disclosure status to other individuals other than the primary caregiver, medication regimen and study condition (whether assigned to the treatment or control condition).

### Analysis procedures

All analyses were conducted using STATA version 15. Bivariate analyses were conducted on baseline sociodemographic and household characteristics, adherence self-efficacy and family support factors. Baseline results were compared between the control and treatment conditions (Table 1). Multilevel analysis was used to examine the relationship between adherence self-efficacy and family support factors over time based on the formula:

$$ \mathrm{yij}=\upbeta 0+{\upbeta}_1{\mathrm{X}}_1\mathrm{ij}+{\upbeta}_2{\mathrm{X}}_2\mathrm{ij}+{\upbeta}_3{\mathrm{X}}_3\mathrm{ij}\cdots +{\upbeta}_{\mathrm{p}}{\mathrm{X}}_{\mathrm{p}}\mathrm{ij}+\upxi \mathrm{ij} $$

where β0 is intercept, X2ij through Xpij are covariates and ξij is a residual.

**Table 1:** Baseline analysis of participant’s sociodemographic and household characteristics, family factors and adherence self-efficacy

Variable	Total Sample % (*N* = 702)	Control Condition % (*n* = 344)	Treatment Condition % (*n* = 358)	t test/χ^2^
Age (mean, SD) (min/max:10–16)	12.4 (1.98)	12.3 (1.93)	12.4 (1.97)	0.85
Gender (female child)	56.4	56.1	56.7	0.07
Orphanhood status				0.70
Single orphan	38.04	38.66	38.55	
Double orphan	37.04	35.76	38.27	
Non-orphan	24.36	25.58	23.18	
Primary caregiver				0.133
Biological parent	46.29	43.60	50.00	
Grand parent(s)	27.56	30.63	30.32	
Other relatives	26.16	26.74	20.95	
Household composition (mean, SD)				
Total number of people in the household) (min/max: 2–18)	5.7 (2.6)	5.8 (2.4)	5.7 (2.6)	0.68
Total number of children in the household) (min/max:1–14)	2.3(1.9)	2.4 (1.8)	2.3(2.1)	0.42
Enrolled in school (yes)	87.32%	87.50	87.15	0.89
HIV status disclosure				0.38
Never	32.34	31.40	33.24	
Sometimes	16.67	15.7	17.60	
About half the time	5.98	7.27	4.75	
Most of the time	12.82	14.53	11.17	
Always	32.19	31.10	33.24	
HIV medication regimen				0.091
1 time per day	22.8	26.2	19.6	
2 times per day	53.6	52.2	55.0	
3 times per day	23.5	21.6	25.4	
Family support measures (mean, SD)				
Child-caregiver communication (min/=max:10–50)	21.1 (7.6)	20.8 (7.5)	21.4(7.8)	0.24
Family cohesion (min/max:12–40)	31.8(6.73)	31.43(6.72)	32.07(6.74)	0.22
Perceived child-caregiver support (min/max: 18–70)	46.5(10.31)	46.37(10.42)	46.62(10.21)	0.75
Adherence self-efficacy (min/max: 20–120)	94.28 (23.25)	95.24 (23.5)	93.35 (23.01)	0.28

The models were built with *-mixed command* using *vce* (robust) option in STATA, that has the advantage that the estimated standard errors are valid even if the level-1 errors are heteroskedastic or autocorrelated [45]. This method was used to study adherence self-efficacy over time (time - level 1), person – level 2 characteristics (family support factors) that influence initial adherence self-efficacy (intercept) and change over time (slope). This approach did not require individuals to have equal numbers of measures and can handle both time invariant and time-varying covariates.

First, the analysis started with building unconditional three-level model to determine between-person and within-person variability in adherence self-efficacy scores (Table 2). Second, level-1 family factor variables (cohesion, communication and support) were added to the unconditional model (Table 3). Like for many other psychological constructs that lack a clearly interpretable or meaningful zero-point, [46] we used Centered Within Cluster (within subject) variables to establish a useful zero point [47]. To accommodate possible endogenous time-varying covariates that are correlated with the random-intercept ζj, the model was fitted allowing for different within and between effects for time-varying covariates by including both subject-mean centered and the occasion-specific deviations from the subject means as the subject-mean centered covariates are uncorrelated with ζj by construction, and the corresponding coefficients could be consistently estimated [45]. Finally, we fitted the model with all the control variables (Table 4). Statistical significance was determined at the 5% level.

**Table 2:** Unconditional multilevel model

				**95% Confidence Interval**	
**Variable**	**Coef.**	**Std. Err.**	**P > |z|**	**Lower**	**Upper**
Adherence self-efficacy	92.507	0.590	0.000	91.35	93.66
				**95% Confidence Interval**	
**Random-effects Parameters**	**Estimate**	**Std. Err**		**Lower**	**Upper**
Variance of Clinic Random Intercept	2.685	2.817		0.344	20.983
Variance of Child Random Intercept	64.347	8.759		49.279	84.021
Variance of Residuals	391.091	110.117		369.898	413.498

LR test vs. linear models: χ^2^ (2) =154.69, *p* = 0.00

**Table 3:** Multilevel analysis model with participants’ sociodemographic and household characteristics

				**95% Confidence Interval**	
**Variable**	**Coef.**	**Std. Err.**	**P > |z|**	**Lower**	**Upper**
Study group (Treatment)	−0.877	1.108	0.428	−3.049	1.294
Gender (female child)	0.892	0.930	0.338	−0.931	2.716
Age	0.835	0.207	0.000	0.429	1.241
Primary caregiver (ref: Biological Parent)					
Grandparents	0.680	1.177	0.563	−1.627	2.987
Other relatives	2.153	1.160	0.063	−0.120	4.427
Orphanhood status (ref: Both parents are alive)					
Single orphan	0.133	1.100	0.904	−2.023	2.289
Double orphan	0.278	1.427	0.845	−2.518	3.074
Household composition					
Number of adults	−0.132	0.236	0.577	− 0.594	0.330
Number of children	0.187	0.323	0.56	−0.445	0.820
Enrolled in school (yes)	0.122	1.083	0.91	−2.000	2.244
HIV status disclosure (ref: never)					
Sometimes	3.162	0.984	0.001	1.233	5.091
About half the time	1.095	2.026	0.589	−2.876	5.065
Most of the time	1.336	1.131	0.238	−0.881	3.552
Always	5.224	1.046	0.000	3.175	7.273
HIV medication regimen	−0.502	0.606	0.408	−1.690	0.687
Constant	78.82	3.89	0.000	71.192	86.447
Wald test: χ^2^(16, 702) = 84.2, *p* = 0.000					
				**95% Confidence Interval**	
**Random-effects Parameters**	**Estimate**	**Std. Err**		**Lower**	**Upper**
Variance (Clinic)	2.261	2.523		0.254	20.144
Variance (Child ID)	62.742	8.365		48.314	81.478
Variance (Residuals)	389.816	10.741		369.322	411.447
LR test vs. linear models: χ^2^ (2, 702) =100.9, *p* = 0000

**Table 4:** Multilevel analysis full model with participants’ sociodemographic and household characteristics, family factors and adherence self-efficacy

				**95% Confidence Interval**	
**Variables**	**Coef.**	**Std. Err.**	**P > |z|**	**Lower**	**Upper**
Treatment group	−0.673	1.049	0.521	−2.730	1.384
Gender (female child)	0.492	0.904	0.586	−1.280	2.264
Age	0.814	0.206	0.000	0.410	1.219
Primary caregiver (ref: Biological Parent)					
Grandparents	0.675	1.149	0.557	−1.577	2.928
Other relatives	2.238	1.138	0.049	0.006	4.469
Orphanhood status (ref: Both parents are alive)					
Single orphan	0.708	1.068	0.507	−1.385	2.800
Double orphan	1.104	1.387	0.426	−1.615	3.824
Household composition					
Number of adults	−0.156	0.232	0.503	−0.611	0.300
Number of children	0.249	0.318	0.433	−0.375	0.874
Enrolled in school	0.079	1.065	0.941	−2.009	2.167
HIV Status Disclosure (ref: never)					
Sometimes	3.349	0.973	0.001	1.441	5.256
About half the time	1.301	2.004	0.516	−2.626	5.228
Most of the time	1.194	1.118	0.286	−0.997	3.385
Always	4.063	1.042	0.000	2.020	6.106
HIV medication regimen	−0.355	0.598	0.552	−1.527	0.816
Family cohesion	0.397	0.065	0.000	0.270	0.524
Perceived child-caregiver support	0.087	0.046	0.056	−0.002	0.177
Child-caregiver communication	0.118	0.053	0.026	0.014	0.222
Constant	59.945	4.376	0.000	51.370	68.521
Wald test: χ^2^ (19, 702) =177.29, *p* = 0.000					
				**95% Confidence Interval**	
**Random-effects Parameters**	**Estimate**	**Std. Err.**		**Lower**	**Upper**
Variance of Clinic Random Intercept	1.809	2.198		0.167	19.561
Variance of Child Random Intercept	53.995	7.793		40.691	71.650
Variance of Residuals	384.261	10.587		364.061	405.581

## Results

Baseline sociodemographic and household characteristics of the sample are presented in Table 1. The average age was 12.4 years and the majority of participants were female (56.4%). About 38% of participants identified as single orphans, meaning they had one surviving biological parent, 24.4% were non-orphans. The majority of adolescents (46.3%) identified a surviving biological parent as the primary caregiver. Participants lived in household with an average of 5.7 people and 2.3 children under the age of 18. At baseline, 87.3% of participants were enrolled in school. In terms of HIV disclosure, 32.3% of adolescents reported that they never keep their HIV status a secret. However, about the same number of adolescents (32.2%) reported that they always keep their HIV a secret. The majority of participants (53.7%) had to take their HIV medication at least twice a day. In terms of family support factors, participants reported moderate levels of communication with their caregivers (mean = 21.1, SD = 7.6), and perceived child-caregiver support (mean = 46.5, SD = 10.3). Higher levels of family cohesion were reported, with mean score of 31.8 (SD = 6.7). Adolescents reported higher levels of adherence self-efficacy, with an average score of 94.28 (SD = 23.25). No statistically significant differences were observed between the control and treatment conditions.

Table 2 presents results from a multilevel unconditional model conducted to determine between-person and within-person variability in adherence self-efficacy scores. The variance in adherence self-efficacy over time for each child was 391.7 (s^2^ = 391.7), explaining 83% of total variance. The variance of the true self-efficacy averaged over time was 79.3(16.7% of variance), while variability of clinic means around the grand mean was only 2.75 (0.6% of the variance). The intraclass correlation for individual level (level 2) was 0.173, and - 0.006 at the clinic level (level 3). The likelihood ratio test indicated that there was evidence that using a random intercept model could explain the variance in adherence self-efficacy even in the absence of any covariates (LR test vs. linear model: χ^2^ (1) = 153.04, *p* = 0.000). Further analysis showed that there was no significant difference between 3-level and 2-level models (LR χ^2^ (1) =1.65, *p* = 0.198), thus analysis was continued with exploring 2-level models.

Table 3 presents results from the multilevel model with socio-demographic and household characteristics. Participants’ age was statistically associated with adherence self-efficacy (*β* = 0.835, *p* = 0.000). In addition, HIV status disclosure, whether sometimes (*β* = 3.16, *p* = 0.001) or always (*β* = 5.22, *p* = 0.000), was also associated with adherence self-efficacy. Group assignment (whether control or treatment condition) was not significant (*β* = − 0.877, *p* = 0.428). The overall model was statistically significant (*F* (16, 702) = 84.2, *p* = 0.000). The variability of self-efficacy over time for each child slightly decreased to 389.8, from 391.1 (Table 2) and variance of the true self-efficacy averaged over time also slightly decreased to 62.7 from 64.3 (Table 2).

Table 4 presents results from the full multilevel analysis models conducted to determine the association between family factors and adherence self-efficacy, controlling for sociodemographic and household characteristics. Family cohesion (*β* = 0.397*, p* = 0.000) and child–caregiver communication (*β* = 0.118, *p* = 0.026) were both positively associated with adherence self-efficacy. Perceived child-caregiver support was not statistically significant (*β* = − 0.087, *p* = 056). The entire model was statistically significant (*F* (19, 702) = 177.29, *p* = 0.000).

In comparison to the previous model with socio-demographic and household characteristics only (Table 3), the variability in adherence self-efficacy changed even more. Specifically, over time, self-efficacy for each child decreased even more to 384.3while variance of the true self-efficacy averaged over time decreased to 53.9 indicating that family factors can play significant role to define adolescents’ adherence self-efficacy.

## Discussion

This study examined the family factors (family cohesion, child-caregiver communication and perceived child-caregiver support) associated with ART adherence self-efficacy among HIV-infected adolescents in southern Uganda. Findings from our study indicate that family cohesion and communication represent emotional support from family members towards HIV-infected adolescents and is felt by adolescents through daily communication and care expressed by family members. Although adolescence involves developing independence as a developmental stage, the supportive relationships and connections with family and caregivers continue to be protective factors. Specifically, for HIV-infected adolescents, family is the primary source of financial, physical, and emotional support. Moreover, most adolescents rely on their family members, especially caregivers, to access HIV care and help administer medication regimes [48, 49]. As such, family members’ assistance in helping with HIV-related concerns or problems encountered by adolescents can contribute to improving adolescents’ self-efficacy in integrating treatment plans into their daily routines. This finding underscores the protective role of family and provides further evidence for transitioning from individual-based to family-focused health interventions.

Orphanhood status was not associated with adherence self-efficacy. This finding somewhat deviates from past research in other sub-Saharan African countries, which showed that orphaned children were more likely to be ART nonadherent [50, 51]. One possible explanation for this finding could be that given that all adolescents included in this study were living within a family (either with a surviving biological parent or another relative), they were already being supported by family members to access and adhere to their medication, regardless of their biological relatedness. This finding also points to the fact that extended family networks in Uganda are very supportive, which is in line with the cultural norms where the extended family is expected to assume caregiving responsibilities following parental death [42].

Lastly, this study substantiates for the reasonable application of adherence self-efficacy concept and social cognitive theory in ART adherence. The study findings respond well to the reciprocal determinism of social cognitive theory and highlight family cohesion and child-caregiver communication as significant environments for improving ART adherence among HIV-infected adolescents in low-resource communities.

Even with these findings, a few limitations are worth pointing out. With a general aim to explore the outcomes of the Suubi+Adherence study, this study examined adherence self-efficacy rather than participants’ ART adherence. Theoretically, adherence self-efficacy is an important determinant of ART adherence behavior, however, it may not reflect the actual behavioral outcome [52]. In addition, family is an emotional unit that profoundly influences its members’ thoughts and actions, but the dynamics between individuals and their family systems are complicated. Although we examined family cohesion and child–caregiver relationship in this study, further research using systems thinking to demonstrate how the Suubi+Adherence project can contribute to adherence self-efficacy improvement through enhancing protective functions of family is needed.

## Conclusion

Study findings underscore family as a microsystem for HIV-infected adolescents in southern Uganda, that provides both tangible and emotional support to enhance adherence self-efficacy. Family cohesion, a composite concept representing family closeness and functioning, as well as communication, are both positively associated with adherence self-efficacy. Hence, our findings provide further evidence to consider including families—biological and extended—and targeting family cohesion in interventions aiming at increasing ART adherence among HIV-infected adolescents, especially in sub-Saharan Africa.

## Data Availability

The datasets analyzed during the current study are available from the corresponding author on reasonable request.

## Abbreviations

ART: Antiretroviral therapy
PLWH: People living with HIV
